# Serotonin Transporter Genotype Linked to Adolescent Substance Use Treatment Outcome through Externalizing Behavior

**DOI:** 10.3389/fped.2014.00071

**Published:** 2014-07-07

**Authors:** Tammy Chung, Jack R. Cornelius, Christopher S. Martin, Robert Ferrell, Stephen A. Maisto, Duncan B. Clark

**Affiliations:** ^1^Western Psychiatric Institute and Clinic, University of Pittsburgh Medical Center, Pittsburgh, PA, USA; ^2^Department of Human Genetics, University of Pittsburgh, Pittsburgh, PA, USA; ^3^Department of Psychology, Syracuse University, Syracuse, NY, USA

**Keywords:** adolescent, alcohol, externalizing behavior, genetics, marijuana, serotonin transporter

## Abstract

Meta-analyses suggest that the serotonin transporter linked polymorphic region (5-HTTLPR) short (S) allele, relative to the long (L) allele, is associated with risk for alcohol dependence, particularly among individuals with early onset antisocial alcoholism. Youth in substance use treatment tend to show antisocial or externalizing behaviors, such as conduct problems, which predict worse treatment outcome. This study examined a pathway in which 5-HTTLPR genotype is associated with externalizing behavior, and the intermediate phenotype of externalizing behavior serves as a link between 5-HTTLPR genotype and substance use treatment outcome in youth. Adolescents (*n* = 142) who were recruited from addictions treatment were genotyped for 5-HTTLPR polymorphisms (S and L_G_ carriers vs. L_A_L_A_), assessed for externalizing and internalizing behaviors shortly after starting treatment, and followed over 6-months. 5-HTTLPR genotype was not associated with internalizing behaviors, and was not directly associated with 6-month substance use outcomes. However, 5-HTTLPR genotype was associated with externalizing behaviors (S and L_G_ > L_A_L_A_), and externalizing behaviors predicted alcohol and marijuana problem severity at 6-month follow-up. Results indicated an indirect (*p* < 0.05) and non-specific (i.e., both alcohol and marijuana severity) effect of 5-HTTLPR genotype on youth substance use treatment outcomes, with externalizing behaviors as an important linking factor. Adolescents in substance use treatment with low expressing (S and L_G_) 5-HTTLPR alleles and externalizing behavior might benefit from intervention that addresses serotonergic functioning, externalizing behaviors, and substance use to improve outcomes.

## Introduction

Serotonergic functioning has been associated with externalizing behaviors, such as conduct problems and aggression; internalizing behaviors, such as depression and anxiety (1); and substance use (2). Externalizing behaviors commonly precede and predict adolescent substance use (3). Internalizing behaviors also have been linked with youth substance use, under certain conditions (4). In adolescents, serotonin transporter genotype may be more strongly associated with intermediate phenotypes of externalizing and internalizing behaviors, than with a substance use phenotype, because the intermediate phenotypes often manifest prior to substance use behavior. Among adolescents in substance use treatment, externalizing behaviors robustly predict worse outcomes (5). Based on these observations, this study of adolescent substance users tested a pathway in which serotonin transporter genotype is associated with externalizing and internalizing behaviors as intermediate phenotypes, and the intermediate phenotypes provide a link between serotonin transporter genotype and substance use treatment outcomes. Increased understanding of how a genetic marker is associated with treatment outcome could ultimately help to guide the development of personalized interventions (6).

Serotonergic functioning involves *SLC6A4*, a gene in the regulatory pathway for serotonin (5-HT), which encodes the 5-HT transporter (5-HTT) protein. The SLC6A4 gene has a functional polymorphism in its regulatory region, known as the 5-HTT linked promoter region (5-HTTLPR), which has two allele variants: a 44-bp insertion [long (L) allele] or deletion [short (S) allele]. The S-allele is associated with a 2- to 2.5-fold decrease in 5-HTT transcription rate compared to the L-allele (7). The L-allele can be further characterized by the presence of an adenine to guanine (A > G) change (8, 9). The L-allele with guanine (L_G_) and the S-allele have similar transcriptional activity, whereas the L-allele with adenine (L_A_) is associated with increased transcriptional activity (10). Based on the functionality of alleles (10), studies have compared “low” vs. “high” expressing allele carriers (i.e., L_A_L_A_ vs. S and L_G_ carriers) (11, 12).

The 5-HTTLPR short, relative to the long, allele has been associated with risk for both externalizing and internalizing behaviors (1, 13). Although most studies report that the 5-HTTLPR S-allele increased risk for externalizing behaviors [e.g., Ref. (14–16)], one study reported no association with conduct problems (17). In adolescents, 5-HTTLPR S-allele played a role in the association between internalizing behaviors and alcohol use, possibly through shared risk factors, such as stress reactivity [e.g., Ref. (4)]. There is stronger evidence of an association between serotonin transporter genotype and externalizing, than internalizing, behaviors. Mixed findings for externalizing behaviors may be due, in part, to the need to account for high vs. low expressing 5-HTTLPR L-allele subtypes (L_A_ and L_G_).

With regard to substance use, the S-allele was associated with slightly increased odds of alcohol dependence in two meta-analyses (18, 19). Importantly, the S-allele was more common among individuals with an early onset, severe form of alcoholism associated with antisocial features (18). Further, a study of adolescents found that the S-allele was associated with the early development of alcohol use [e.g., Ref. (20)]. These findings suggest that the association between 5-HTTLPR genotype and alcohol involvement may occur through a pathway that involves externalizing behavior as an intermediate phenotype (1, 16, 21).

Few studies have examined 5-HTTLPR genotype in relation to substance use treatment outcome. In a study of African American cocaine and alcohol abusers, S-allele homozygotes showed less improvement on drinking measures at 6-month follow-up (22). Another study found that the 5-HTTLPR S-allele was associated with relapse to alcohol over 3-month follow-up among Caucasian males treated for alcohol dependence (23). The study of treated alcohol dependent males discussed the possibility that the 5-HTTLPR S-allele does not directly influence substance use relapse, but may have effects on outcome through an intermediate phenotype involving impaired behavioral control, as manifested by externalizing behaviors. No study has examined whether 5-HTTLPR genotype has an indirect effect on treatment outcome through intermediate phenotypes of externalizing or internalizing behaviors.

**Figure 1:**
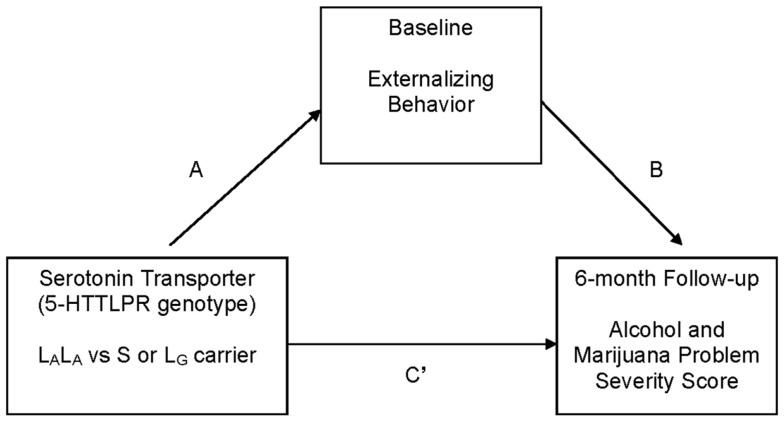
**Hypothesized mediation (indirect effects) model that was tested**. C′ represents the direct effect of independent variable (5-HTTLPR genotype: high vs. low expressing) on the dependent variable (alcohol and marijuana problem severity at 6 months) after controlling for the intervening variable (externalizing behavior). The C path (not labeled in the figure) represents the total effect of the independent variable on the dependent variable. Covariates: gender, age, race (Caucasian vs. all other ethnicity), socio-economic status, and baseline frequency of substance use.

This study addresses gaps in knowledge regarding 5-HTTLPR genotype as a predictor of adolescent substance use treatment outcome. We hypothesized, as suggested by research with treated adults (23), that 5-HTTLPR genotype would be indirectly associated with adolescent posttreatment alcohol and marijuana problem severity through the intermediate phenotype of externalizing behavior. Specifically, S/L_G_ allele carriers, relative to L_A_ homozygotes, were predicted to report greater externalizing behavior [see Ref. (24)], and greater externalizing behavior was predicted to be associated with greater alcohol and marijuana problem severity at 6-month follow-up [see Ref. (5)] (see Figure 1).

## Materials and Methods

### Participants

In the United States, 6.1% of youth (ages 12–17) met criteria for a DSM-IV alcohol or illicit drug use disorder in the past year (25). The prevalence of past year substance use disorder in the urban setting of Pittsburgh, PA, USA is similar to national prevalence rates (26). This study recruited 209 adolescents (ages 14–18) from community-based substance use treatment in Pittsburgh. Of the 209 youth, 153 (73.2%) youth were successfully genotyped (*n* = 55 did not provide DNA, *n* = 1 sample could not be genotyped). Cases with missing data due to incomplete baseline questionnaires (*n* = 11) were excluded, resulting in a baseline analysis sample of 142 adolescents. Youth who were vs. were not included in the analysis sample did not differ (*p* > 0.10) on demographic characteristics (age, gender, race, and SES) or frequency of using alcohol or marijuana at baseline.

The analysis sample was 66.2% male. Based on self-reported race/ethnic identity, the sample was 86.6% Caucasian, 7.0% African American, and 6.3% other ethnicity (e.g., bi-racial). Average age was 16.7 (SD = 1.2). Sample demographic characteristics (Table 1) were similar to those of youth admitted to publicly funded substance use treatment (27). Participants were, on average, middle-class in socio-economic status [mean = 2.4, SD = 1.1; range = 1–5; (28)]. Most (85.9%) had a current (past 6 months) DSM-IV cannabis use disorder, and almost half (47.2%) had a current DSM-IV alcohol use disorder. Six-month follow-up data were available for 89% (*n* = 126) of the sample; those who did vs. those who did not complete follow-up did not differ on demographics, internalizing or externalizing symptoms, and baseline alcohol or marijuana problem severity (*p* > 0.15).

**Table 1:** **Descriptive statistics for the total sample, and high vs. low expressing allele groups**.

	Total		High exp		Low exp	
Demographics	*n*	%	*n*	%	*n*	%
Female	48	33.8	11	31.4	37	34.6
Male	94	66.2	24	68.6	70	65.4
Ethnicity
Caucasian	123	86.6	28	80.0	95	88.8
African American	10	7.0	3	8.6	7	6.5
Multi-racial	9	6.3	4	11.4	5	4.7

**Baseline**	**Mean (SD)**		**Mean (SD)**		**Mean (SD)**	

Age	16.7 (1.2)		16.7 (1.2)		16.7 (1.2)	
Socio-economic status (28)	2.5 (1.1)		2.5 (1.2)		2.5 (1.1)	
Externalizing composite *T* score	62.1 (13.4)		57.2 (13.5)		63.7 (13.0)	
Internalizing composite *T* score	52.1 (13.4)		49.1 (14.6)		53.2 (12.9)	
Frequency of substance use (past 6-months)^a^
Alcohol use	3.6 (1.9)		3.3 (1.9)		3.7 (1.9)	
Marijuana use	5.6 (2.5)		5.6 (2.8)		5.7 (2.3)	

	***n***	**%**	***n***	**%**	***n***	**%**

Current DSM-IV alcohol use disorder	67	47.2	13	37.1	54	50.5
Alcohol abuse	54	38.0	11	31.4	43	40.2
Alcohol dependence	13	9.2	2	5.7	11	10.3
Current DSM-IV cannabis use disorder	122	85.9	27	77.1	95	88.8
Cannabis abuse	73	51.4	20	57.1	53	49.5
Cannabis dependence	49	34.5	7	20.0	42	39.3
Current DSM-IV psychopathology
Conduct disorder	53	37.3	13	37.1	40	37.4
Attention deficit hyperactivity disorder	50	35.2	7	20.0	43	40.2
Major depression	20	14.1	7	20.0	13	12.1

**6-month Follow-up (time frame: past 6 months)**	**Mean (SD)**		**Mean (SD)**		**Mean (SD)**	

Frequency of substance use^a^
Alcohol use	2.9 (1.8)		2.4 (1.8)		3.0 (1.9)	
Marijuana use	3.6 (2.7)		2.9 (2.4)		3.8 (2.8)	
Rutgers alcohol problems index score	7.9 (10.3)		8.6 (11.5)		10.1 (12.2)	
Rutgers marijuana problems index score	11.1 (13.4)		8.5 (11.3)		14.8 (16.4)	

*Baseline *N* = 142; 6-month *n* = 126; high exp, high expressing (L_A_L_A,_*n* = 35); low exp, low expressing (S and L_G_, *n* = 107); BL, baseline; SD, standard deviation; current, past 6 months*.

*^a^Frequency coded: 0 = never used, 1 = no use in the last 6 months, 2 = used < once per month, 3 = used once per month, 4 = used 2–3 times per month, 5 = used once per week, 6 = used 2–3 times per week, 7 = used 4–6 times per week, and 8 = daily use*.

### Procedure

Youth admitted to community-based intensive outpatient substance use treatment were approached to participate in a longitudinal study on treatment outcome (29, 30). Treatment involved three 3-h group sessions per week for 6–8 weeks, with content that supported a goal of abstinence from alcohol and illicit drugs. Informed consent (from 18 years olds) or assent (from minors, with informed consent for the minor’s participation provided by the minor’s parent) was obtained prior to initiating study procedures. The baseline assessment, which was typically completed within 2 weeks of treatment entry, collected substance use and psychiatric data. The same domains were assessed at follow-up. Baseline data were collected from 2004 to 2009. Highly trained research associates collected interview data with high reliability (31), and saliva DNA according to protocol. Youth completed questionnaires at home, which were returned at the interview session or by mail. The University of Pittsburgh’s Institutional Review Board approved the study protocol (no. IRB0402001).

### DNA collection and genotyping

DNA from saliva was collected using a mouthwash protocol (32). Samples were subjected to whole genome amplification using multiple displacement amplification (33), quantified by the pico green protocol, and diluted to 40 ng/μl for storage. A polymerase chain reaction protocol followed by double restriction endonuclease digestion was used to identify the 5-HTTLPR (*SLC6A4*) and rs25531 variants: S, L_A_, and L_G_ (9). The primer sequences were: (forward) 5′-TCCTCCGCTTTGGCGCCTCTTCC-3′, and (reverse) 5′-TGGGGGTTGCAGGGGAGATCCTG-3′. The L-allele was subtyped for rs25531. The A > G SNP of rs25531 was concurrently detected by digesting the amplified fragments with *Msp*I (New England Biolabs, Beverly, MA, USA), where the A > G substitution creates an additional *Msp*I site. Amplification products were simultaneously resolved by electrophoresis on 3.5% agarose gels.

The genotype distribution in the total sample (*N* = 142) was: SS, *n* = 19 (13.4%); SL_G_, *n* = 9 (6.3%); L_G_L_G_, *n* = 2 (1.4%); SL_A_, *n* = 60 (42.3%); L_G_L_A_, *n* = 17 (12.0%); and L_A_L_A_, *n* = 35 (24.6%). SS, SL, and LL frequencies did not deviate significantly from Hardy–Weinberg equilibrium in the total sample (χ^2^[df = 1] = 0.17), or in the Caucasian subsample^1^ (*N* = 123: SS *n* = 18, SL *n* = 65, LL *n* = 40; χ^2^[df = 1] = 1.04). As in prior studies [see Ref. (8)], “low” expressing S and L_G_ alleles were grouped together and compared with “high” expressing L_A_ allele (total sample: L_A_L_A_ *n* = 35 vs. all other types *n* = 107; Caucasian: L_A_L_A_ *n* = 28 vs. all other types *n* = 95).

### Measures of substance involvement and psychopathology

An adapted structured clinical interview for DSM-IV SUDs [SCID; (34)] assessed past 6-month SUD diagnoses at baseline and follow-up. The adapted SCID has acceptable reliability and validity (31). The Kiddie-schedule for affective disorders and schizophrenia (35) assessed current DSM-IV psychopathology.

The youth self-report (YSR; ages 14–17, 112 items) or young adult self-report (YASR; age 18, 116 items) (36, 37) was completed at baseline. Items used a 6-month time frame, with responses coded as “not true,” “sometimes true,” or “often true.” A normalized *T* score was computed for the internalizing (withdrawn, somatic complaints, and anxious/depressed subscales) and externalizing (delinquent behavior and aggressive behavior subscales) composite indices. The externalizing composite score includes items on substance use (e.g., “I use drugs for non-medicinal purposes”).

The drug consumption questionnaire assessed frequency of alcohol and marijuana use in the past 6 months at each assessment using a 9-point scale (0 = never used, 1 = no use in the last 6 months, 2 = used less than once per month, 3 = used once per month, 4 = used 2–3 times per month, 5 = used once per week, 6 = used 2–3 times per week, 7 = used 4–6 times per week, and 8 = daily). The substance use frequency items have satisfactory reliability and validity (31).

The Rutgers alcohol problem index [RAPI; (38)] and Rutgers marijuana problem index [RMPI; (39)], which each include 18 items rated on a 0–4 scale (0 = 0 times, 1 = 1–2 times, 2 = 3–5 times, 3 = 6–10 times, and 4 ≥ 10 times), assessed alcohol and marijuana problem severity at 6-month follow-up (RAPI alpha = 0.91; RMPI alpha = 0.92). RAPI and RMPI scores were positively correlated (*r* = 0.67, *p* = 0.001).

### Data analysis

Bivariate correlations were run as a preliminary step to determine the utility of testing hypothesized mediation (indirect) effect models (40). Tests for indirect effect used a bootstrapping procedure (5,000 resamples) available as an SPSS macro (41). Each model (see Figure 1; Table 2) tested an “A path,” which represents the path from the independent variable (serotonin transporter genotype) to the intervening variable (e.g., externalizing score); a “B path,” which represents the direct effect of the intervening variable (e.g., externalizing score) on the dependent variable (baseline RAPI/RMPI score); a “C path,” which represents the total effect of the independent variable on the dependent variable; and a “C′ path,” which represents the direct effect of the independent variable on the dependent variable, after controlling for the intervening variable. A significant indirect effect in the regression model is indicated when the 95% bias-corrected and accelerated (BCa) confidence interval around the unstandardized coefficient does not include 0 (41). An indirect effect can be detected in the absence of a significant direct effect [e.g., Ref. (40, 42)]. Regression analyses controlled for gender, age, race (i.e., Caucasian vs. other race), socio-economic status, and baseline substance use. There was no difference in the pattern of results using transformed and untransformed variables, so results using untransformed data are reported [see Ref. (43)]. Because 5-HTTLPR allele frequencies differ across race (44), we conducted secondary analyses only in the Caucasian subsample.

**Table 2:** **Parameter estimates for models, testing indirect effects of externalizing symptoms on the association between serotonin transporter genotype (A/G) and 6-month RAPI and RMPI score**.

	*B*	SE	*t*	*p*
**OUTCOME: 6-month RAPI**				
A path	6.69	2.60	2.57	0.011
B path	0.24	0.07	3.46	0.001
C path	1.33	2.07	0.64	0.523
C′path	−0.29	2.03	−0.14	0.885
Covariates: gender	3.28	1.83	1.79	0.076
Age	−0.34	0.73	−0.46	0.642
Race	0.06	2.69	0.02	0.983
SES	1.35	0.81	1.66	0.098
BL alcohol days	1.48	0.46	3.21	0.002

*Indirect effect point estimate = 1.62 (BCa 95% CI: 0.36, 3.84)*.				
*Model summary: n* = *125; R*^2^ = *0.19, F(7, 117)* = *4.03, p* = *0.0005*.				
**OUTCOME: 6-month RMPI**				
A path	6.44	2.66	2.42	0.017
B path	0.28	0.09	3.09	0.002
C path	4.88	2.71	1.80	0.075
C′path	3.05	2.68	1.13	0.259
Covariates: gender	6.07	2.46	2.47	0.015
Age	0.09	0.94	0.10	0.922
Race	1.86	3.46	0.54	0.592
SES	1.35	1.05	1.28	0.202
BL marijuana days	1.20	0.46	2.58	0.011

*Indirect effect point estimate* = *1.83 (BCa 95*% *CI: 0.39, 4.17)*.				
*Model summary: n* = *122; R*^2^ = *0.20, F(7, 114)* = *4.18, p* = *0.0004*.				

*BL, baseline; 6-mo, 6-month follow-up; RAPI, Rutgers alcohol problems inventory; RMPI, Rutgers marijuana problems inventory; alcohol/marijuana days, number of use days in the month prior to BL; *B*, unstandardized coefficient; SE, standard error; gender (0 = female, 1 = male); race (0 = other race/ethnicity, 1 = Caucasian); SES, socio-economic status (1–5, 1 = high SES). A path, independent variable (serotonin transporter genotype: 0 = L_A_L_A_, 1 = S/L_G_ carrier) to intervening variable (externalizing score); B path, direct effect of intervening variable (externalizing score) on dependent variable (6-month RAPI/RMPI score); C path, total effect of independent variable on dependent variable; C′ path, direct effect of independent variable on dependent variable, after controlling for the intervening variable. Covariates: partial effect of covariates on dependent variable*.

## Results

### Comparison of high vs. low expressing allele groups

In the total sample (see Table 1 for descriptives), 5-HTTLPR genotype (0 = L_A_L_A_, 1 = S or L_G_ allele carrier) was not associated with a current DSM-IV alcohol, marijuana, major depression, or conduct disorder diagnosis at baseline (*p* = 0.17, 0.09, 0.25, and 0.98, respectively). However, the low, compared to high, expressing allele group was more likely to have current DSM-IV ADHD at baseline (χ^2^ = 4.71, df = 1, *p* < 0.05; also observed in only the Caucasian subsample: χ^2^ = 5.06, df = 1, *p* < 0.05). High vs. low expressing genotype did not differ on internalizing score (*p* > 0.10), or frequency of alcohol use (at baseline *p* > 0.20 or 6-month follow-up *p* > 0.10) and marijuana use (at baseline *p* > 0.75 or 6-month follow-up *p* > 0.09). However, the high, relative to low, expressing genotype had a higher externalizing score (*t* = −2.56, df = 140, *p* = 0.01; also observed in only the Caucasian subsample: *t* = −2.33, df = 121, *p* = 0.02).

### Testing an indirect pathway linking 5-HTTLPR genotype and treatment outcome

Preliminary bivariate correlations supported a possible mediation (“indirect”) pathway involving an association between 5-HTTLPR low vs. high expressing genotype and externalizing behavior (*r* = 0.21, *p* = 0.01), and externalizing behavior with 6-month alcohol and marijuana-related problems (RAPI *r* = 0.30, *p* < 0.01; RMPI = 0.27, *p* < 0.01). These correlations held in the Caucasian subsample (*p* < 0.05). Thus, indirect effect analyses focus on the association between 5-HTTLPR genotype, externalizing behavior, and 6-month RAPI and RMPI scores (see Figure 1).

In predicting 6-month RAPI score (Table 2), 5-HTTLPR genotype was associated with externalizing behavior (*B* = 6.69, *p* = 0.01; low expressing group was associated with greater externalizing behavior), and externalizing behavior was positively associated with 6-month RAPI score (*B* = 0.24, *p* = 0.001). 5-HTTLPR genotype was not directly associated with 6-month RAPI (*B* = 1.33, *p* = 0.52). A significant indirect effect linking 5-HTTLPR genotype to 6-month RAPI scores through externalizing behavior, adjusting for covariates, was observed: point estimate = 1.62 (BCa 95% CI: 0.36, 3.84).

For the model predicting 6-month RMPI score, the low expressing group was associated with greater externalizing behavior (*B* = 6.44, *p* = 0.02), and externalizing behavior was positively associated with 6-month RMPI score (*B* = 0.28, *p* = 0.002). 5-HTTLPR genotype was not directly associated with 6-month RMPI (*B* = 4.88, *p* = 0.08). An indirect effect of externalizing behavior in the association between 5-HTTLPR genotype and 6-month RMPI score was observed: point estimate = 1.83 (BCa 95% CI: 0.39, 4.17).

Analyses of the Caucasian subsample, adjusting for covariates, yielded similar results regarding indirect effects of externalizing behavior score in the association between 5-HTTLPR genotype and 6-month RAPI and RMPI scores: RAPI point estimate = 1.66 (BCa 95% CI: 0.34, 4.25); RMPI point estimate = 1.98 (BCa 95% CI: 0.39, 4.79).

## Discussion

Results supported the hypothesis that low, relative to high, expressing 5-HTTLPR genotype would be associated with greater externalizing behavior among youth in substance use treatment. Further, 5-HTTLPR genotype was associated with treatment outcome only indirectly, through externalizing behavior. These results are applicable mainly to Caucasian youth in substance use treatment. The finding that the direct association between 5-HTTLPR genotype and 6-month alcohol and marijuana problem severity was not significant, suggests that externalizing behaviors provide an important link in the pathway between 5-HTTLPR genotype and 6-month substance use treatment outcome in this adolescent sample.

The association between 5-HTTLPR genotype and externalizing behaviors in an adolescent treatment sample is consistent with the stronger association between low expressing 5-HTTLPR genotype and alcoholism among adults with early onset, antisocial alcoholism (i.e., type II) (18), and with externalizing behaviors more generally (45). In this adolescent sample, the stronger association between 5-HTTLPR genotype and externalizing behavior, relative to a substance use phenotype (e.g., DSM-IV alcohol use disorder), may reflect that the sample has not passed through young adulthood, a period of high risk for the onset of substance use disorder.

The absence of an association between 5-HTTLPR genotype and internalizing behaviors in this study may reflect the relatively low prevalence of mood and anxiety disorders in this sample. Further, the association of ADHD with low expressing alleles observed in this study differs from other work, which reported associations between the L-allele and ADHD [e.g., Ref. (46)]. The disparate findings obtained in this study relative to prior research might be explained, for example, by specific patterns of co-occurring psychopathology in a given sample, interactions among genes (e.g., epistasis) and haplotypes [e.g., Ref. (47)], and effects of environmental conditions that were not examined.

Results are generally consistent with adult treatment studies, which found that the 5-HTTLPR S-allele was directly associated with worse outcome (22, 23). The current study provides a unique contribution in finding that low expressing 5-HTTLPR alleles were only indirectly linked with alcohol- and marijuana-related problems at 6-month follow-up in treated youth, through severity of externalizing behavior. These novel results in a sample of youth in substance use treatment tentatively suggest that 5-HTTLPR genotype, which was associated with externalizing behavior, might provide a possible target for treatment related to serotonergic functioning and externalizing behavior [see Ref. (48)].

The mixed findings in the literature regarding associations of low expressing 5-HTTLPR genotypes with externalizing and substance use phenotypes urge caution in the interpretation of this study’s results. Although study findings are consistent with research reporting that low, relative to high, expressing alleles are associated with externalizing behavior and substance use, and other studies have found the opposite pattern. For example, low level of alcohol response in men, which is associated with alcohol dependence, was correlated with high expressing 5-HTTLPR genotypes (L_A_ homozygotes) (8), suggesting that an association between the L-allele and alcohol dependence may be specific to certain subgroups (18), and differ by gender (49). The mixed findings across studies might reflect differences in sample ascertainment and phenotype definition, and whether co-occurring psychiatric conditions, and L-allele subtypes and haplotypes were examined (50).

This study had limitations. The majority of participants were Caucasian, male, and in substance use treatment (primarily for cannabis use disorder), which limits generalizability. Sample size overall was limited. Analyses did not correct for multiple comparisons, and results require replication. A comparison group of adolescents with no substance use problems was not examined. Measures of more narrowly defined phenotypes (e.g., response inhibition), which may underlie externalizing behaviors, were not examined. Haplotype analyses were not done. Only one candidate region of the SLC6A4 gene was examined. Ideally, whole exome sequencing of all genes involved in serotonergic metabolism would be conducted to rule out rare variants, which could bias results by exerting a stronger effect on the phenotype than HTTLPR length alone.

Youth with low expressing 5-HTTLPR genotype and externalizing behaviors may benefit from interventions that address serotonergic functioning [see Ref. (48)], and intensive treatment that simultaneously aims to reduce externalizing behaviors and substance use [e.g., Ref. (51)]. However, further research is needed to more precisely identify relevant treatment targets, and to replicate results. With regard to public health implications, predictive and diagnostic genetic testing for psychiatric conditions is promising, yet premature, given mixed findings and relatively small effects of common genetic variants (52). However, greater understanding of single gene effects, based on multiple methods (e.g., whole exome sequencing, model systems) and converging results, is critical to characterizing the pathophysiology of complex diseases, which could ultimately guide the development of novel treatments for substance use (6).

## Conflict of Interest Statement

The authors declare that the research was conducted in the absence of any commercial or financial relationships that could be construed as a potential conflict of interest.

## Footnotes

^1^The genotype distribution in the Caucasian sample (*N* = 123) was: SS, *n* = 18 (14.6%); SL_G_, *n* = 9 (7.3%); L_G_L_G_, *n* = 2 (1.6%); SL_A_, *n* = 56 (45.5%); L_G_L_A_, *n* = 10 (8.1%); and L_A_L_A_, *n* = 28 (22.8%).
